# Association between spasticity of the hip and development of hip displacement in children: a cohort study of 786 hips

**DOI:** 10.2340/17453674.2026.46169

**Published:** 2026-06-22

**Authors:** Muhammed BAKHTIYAR, Afrim ILJAZI, Anders ODGAARD, Christian WONG, Michael Mørk PETERSEN, Andreas BALSLEV-CLAUSEN

**Affiliations:** 1 Department of Orthopedic Surgery, Rigshospitalet, Copenhagen University Hospital; 2 Department of Orthopedic Surgery, Odense University Hospital, Odense, Denmark

## Abstract

**Background and purpose:**

Hip migration is a common complication in children with cerebral palsy (CP). Although spasticity has long been considered a contributing factor, muscle-specific associations with hip migration remain unclear. We aimed to investigate whether assessed hip spasticity was associated with hip displacement risk in non-ambulant children with CP.

**Methods:**

In this population-based cohort study using Danish Cerebral Palsy Follow-Up Program (CPOP) data, children classified as Gross Motor Function Classification System (GMFCS) levels IV–V with at least 1 hip radiograph were included. Pathological hip migration was defined as migration percentage (MP) ≥ 30%. Spasticity was assessed using the Modified Ashworth Scale (MAS) for hip adductors, flexors, and extensors. Overall hip spasticity was defined as summed MAS (0, 1–3, 4–6, 7–9). Cumulative incidence was estimated with death as a competing event, and associations were evaluated using Fine–Gray regression adjusted for GMFCS level.

**Results:**

422 children (786 hips) were included; 374 hips (48%) reached MP ≥ 30%, and death occurred as a competing event in 16 patients (25 hips). Higher overall hip spasticity was associated with increased risk of hip migration, with subdistribution hazard ratios (sHRs) of 1.46 (CI 1.05–2.01), 1.77 (CI 1.22–2.58), and 2.53 (CI 1.59–4.02) for MAS 1–3, 4–6, and 7–9, respectively, compared with MAS 0. Adductor spasticity showed the most consistent association with sHRs ranging from 1.44 to 1.97 across MAS categories.

**Conclusion:**

In children with CP classified as GMFCS levels IV–V, higher clinically assessed hip spasticity—particularly of the hip adductors—was associated with development of a hip at risk for displacement.

Cerebral palsy (CP) is a common neurodevelopmental disorder arising in infancy or early childhood, with a birth prevalence of approximately 1.6 per 1,000 live births in high-income countries and higher estimates reported globally [1–4]. It is characterized by disorders of movement and posture and is often accompanied by spasticity and muscle contractures, which may affect hip migration. Traditionally, hip migration in CP has been attributed to increased muscle overactivity and progressive soft-tissue tightness around the hip, particularly in the adductors and flexors, promoting a flexion–adduction posture (“spastic hip position”) that alters the hip joint force vector from anteromedial to posterolateral and may contribute to lateral displacement of the femoral head [5–7]. The risk of pathological hip migration with migration percentage (MP) ≥ 30% (Figure 1) increases with higher Gross Motor Function Classification System (GMFCS) level [1]. However, the relative contribution of spasticity in specific muscle groups to the development of hip migration remains incompletely understood.

**Figure 1:**
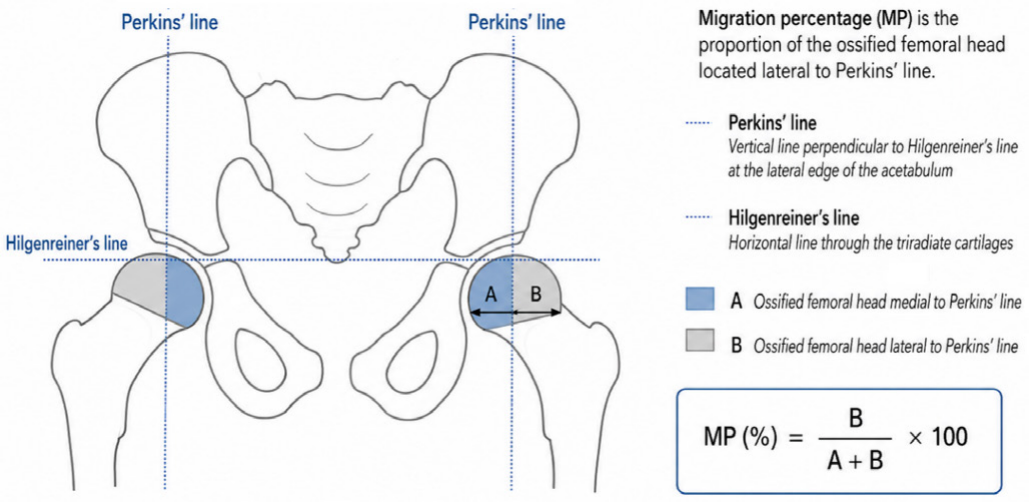
Measurement of migration percentage (MP) according to Reimers.

In clinical practice, spasticity is commonly assessed using the Modified Ashworth Scale (MAS), despite known limitations in inter-rater reliability, particularly in the lower limbs [8,9]. Previous studies of hip migration have primarily focused on gross motor function, ambulation, or developmental milestones as predictors [10–12], whereas the prognostic relevance of clinically assessed, muscle-specific spasticity at the individual hip level remains unclear.

We aimed to investigate the association between clinically assessed hip spasticity and the risk of pathological hip migration, defined as MP ≥ 30%. Specifically, we examined whether overall and muscle-specific hip spasticity were associated with the development of hip migration.

## Methods

### Study design and study population

This was a nationwide register-based cohort study using prospectively collected data from the Danish Cerebral Palsy Follow-Up Program (CPOP), covering children with cerebral palsy from all regions of Denmark between 2008 and December 31, 2023.

Data included standardized clinical evaluations and radiographic hip assessments recorded as part of routine yearly follow-up. The source population included all children enrolled in CPOP from birth until the latest follow-up available.

Children were eligible for inclusion if they were classified GMFCS level IV or V at any time during follow-up and had at least 1 hip radiograph with a measurable MP [1,5]. Restricting the cohort to GMFCS levels IV–V allowed us to focus on non-ambulant children, in whom the risk of pathological hip migration is highest.

The study was conducted and reported in accordance with the STROBE guidelines [13].

### Data source

CPOP is a nationwide follow-up program and clinical quality database for children with CP in Denmark. It includes standardized clinical assessments and radiographic hip surveillance, including registration of gross motor function, muscle tone, passive range of motion, and migration percentage (MP). The structure, variables, quality indicators, and national implementation of CPOP have been described previously [10,14].

### Outcome and exposure

The primary outcome was MP ≥ 30% [15,16], assessed from anteroposterior pelvic radiographs. MP was calculated according to the method described by Reimers, representing the proportion of the ossified femoral head located lateral to Perkins’ line (see Figure 1) [15,17].

For hips reaching MP ≥ 30%, event time was defined as age at the first radiograph meeting this threshold. Hips that did not reach MP ≥ 30% during follow-up were censored at age at the last available radiograph.

Spasticity was assessed clinically using the MAS during routine CPOP examinations. MAS scores were recorded separately for hip adductors, flexors, and extensors for each side. Information on spasticity-targeted treatment, including non-surgical tone-modifying interventions, was not registered in the surveillance program and was therefore not available in the dataset for the analyses.

A landmark approach was applied to define exposure status. For each hip, the most recent MAS assessment recorded prior to the event date (MP ≥ 30%) or censoring was identified and used in the analyses. This ensured that exposure was defined prior to the outcome, although MAS assessments were often obtained in temporal proximity to radiographic follow-up.

MAS scores were categorized into 4 groups: MAS 0, MAS 1 (including 1+), MAS 2, and MAS ≥ 3. Muscle-specific analyses were performed for adductors, flexors, and extensors using the same categorization. MAS was used as the primary tone-related measure in the analyses, with MAS = 0 as the reference category. The available data did not allow separate identification of hypotonia within this category. Overall hip spasticity was defined as the sum of MAS scores for the hip adductors, flexors, and extensors for the corresponding side (range 0–9). Due to sparse data in the highest sums, overall hip MAS was categorized as 0, 1–3, 4–6, and 7–9 for the primary analysis.

GMFCS level (IV or V) was included as a covariate in the regression analyses to account for potential differences in overall functional severity within the non-ambulant population.

### Statistics

Analyses were performed at the hip level, with each hip contributing only once to the analysis. As children could contribute up to 2 hips, robust variance estimation clustered at the child level was applied to account for within-child correlation. Follow-up was defined using age as the underlying time scale, measured from birth until the first occurrence of hip migration (MP ≥ 30%) or censoring, which occurred until a minimum of 25 hips remained at risk.

Hips not reaching MP ≥ 30% during follow-up were censored at the age at the last available radiographic assessment. Death before the occurrence of MP ≥ 30% was treated as a competing event.

Cumulative incidence of hip migration (MP ≥ 30%) was estimated using the Aalen–Johansen estimator, accounting for death as a competing risk. Cumulative incidence curves were generated overall and stratified by spasticity categories.

Associations between spasticity and the risk of hip migration were quantified using Fine–Gray subdistribution hazard models, with death treated as a competing event and adjustment for GMFCS level (IV vs V). Subdistribution hazard ratios (sHR) with 95% confidence intervals (CI) were reported.

The primary analysis examined overall hip spasticity. Secondary analyses evaluated muscle-specific spasticity of the adductors, flexors, and extensors. An exploratory mutually adjusted model including all 3 muscle groups simultaneously was also performed.

For descriptive and illustrative purposes, Kaplan–Meier methods were used to generate supplementary figures, with cumulative incidence expressed as 1 − S(t). These analyses did not account for competing risks and were not used for primary inference. Group differences in passive hip range-of-motion were explored using linear mixed-effects models accounting for clustering at child level.

All analyses were performed using R version 4.5.2 (R Foundation for Statistical Computing, Vienna, Austria).

### Ethics, registration, data sharing plan, funding, and disclosures

The Regional Committee on Health Research Ethics reviewed the study (H-22046940) and determined that, according to the Danish Act on Research Studies, formal ethical approval was not required. We adhered to the relevant regulations and received approval from the local review board Privacy (p-2023-15158). The ELSASS Foundation (22-B01-0670) funded the study. Complete disclosure of interest forms according to ICMJE are available on the article page, doi: 10.2340/17453674.2026.46169

## Results

**Figure 2:**
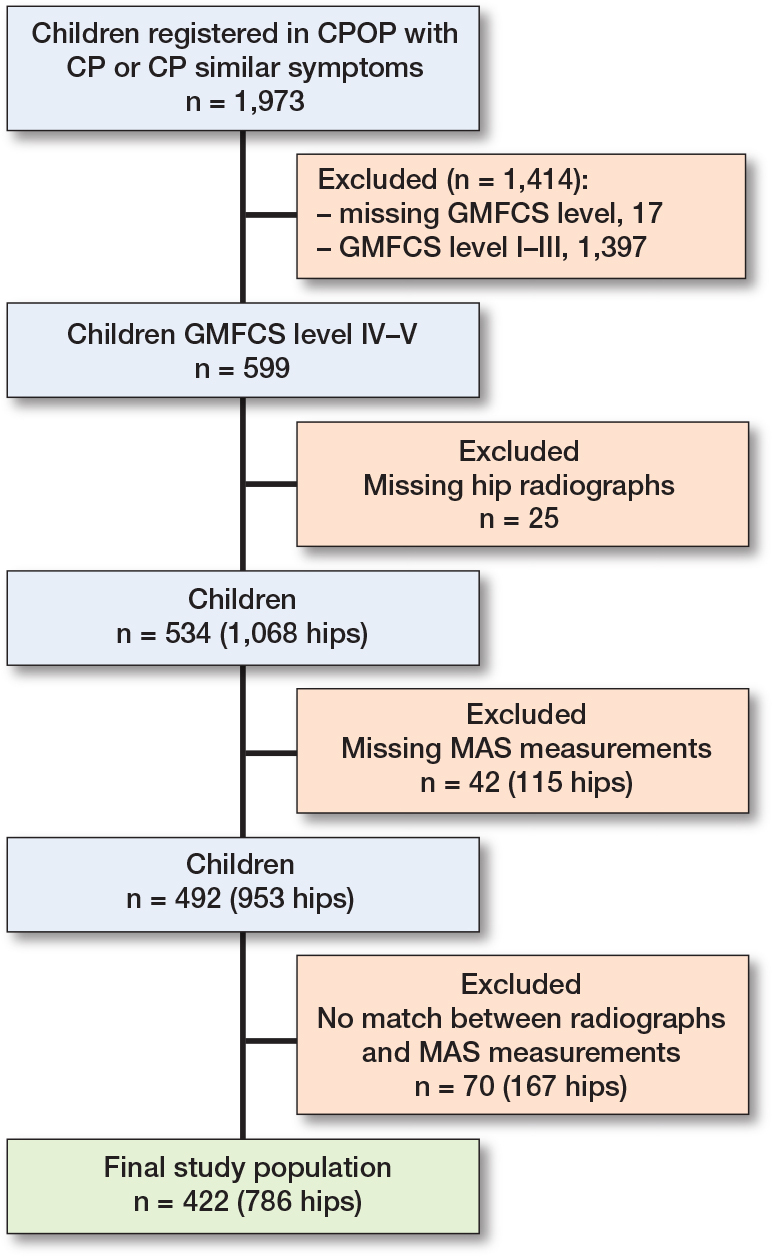
Flowchart of study inclusion. CPOP = the Danish Cerebral Palsy Follow-Up Program; GMFCS = Gross Motor Function Classification System; MAS = Modified Ashworth Scale.

1,973 children were registered in CPOP during the study period. Of these, 559 children were classified as GMFCS level IV or V at any time during follow-up. Among these, 534 (96%) had at least 1 hip radiograph with measurable MP and were eligible for analysis, corresponding to 1,068 hips (Figure 2). After applying the landmark definition of spasticity (most recent MAS assessment prior to event or censoring), 786 hips from 422 children had complete data on overall hip MAS and were included in the primary competing-risk analyses. Follow-up was possible up to 16 years with a minimum of 25 hips remaining at risk and alive. Within this analysis population, hip migration (MP ≥ 30%) occurred in 374 hips, and death occurred as a competing event in 25 hips; the remaining hips were censored at the last available radiograph. The mean age at inclusion, defined as age at first radiographic assessment, was 3.3 (standard deviation [SD] 2.4) years, and the mean radiological follow-up time was 2.3 (SD 2.4) years (Table 1). The median time from MAS assessment to the corresponding radiograph used in the analysis was 43 days (IQR 15–162) overall. Passive hip ROM was similar across outcome groups, except for an overall difference in hip flexion (P = 0.035), with mean values of 124° (SD 18), 121° (SD 20), and 125° (SD 16) in the censored, death, and MP ≥ 30% groups, respectively.

**Table 1:** Baseline characteristics of the study population. Age at inclusion was defined as age at first radiograph. Follow-up was defined as time from first radiograph to first migration percentage (MP) ≥ 30% or last radiograph. Passive hip range of motion was recorded at the same assessment as the MAS. Values are count (%) unless otherwise stated

Characteristic	Values
Children	422
Hips	786
Age at inclusion, years, mean (SD)	3.3 (2.4)
GMFCS level IV	186 (38)
GMFCS level V	306 (62)
Radiographic follow-up, years, mean (SD)	2.3 (2.5)
Hips reaching MP ≥ 30% during follow-up	374 (48)
Children with ≥ 1 hip reaching MP ≥ 30%	251 (60)
Days from MAS assessment to corresponding radiograph, median (IQR)	43 (15–162)
Overall hip MAS group, n hips (%)	
MAS 0	206 (26)
MAS 1	305 (39)
MAS 2	130 (17)
MAS ≥ 3	145 (18)
Passive hip range of motion (SD)	
Abduction	37 (14)
Flexion	125 (17)
Internal rotation	53 (16)
External rotation	52 (15)
Extension	9 (11)

GMFCS = Gross Motor Function Classification System; IQR = interquartile range; MAS = Modified Ashworth Scale; MP = migration percentage (see Figure 2); SD = standard deviation.

### Overall cumulative incidence of MP ≥ 30%

The cumulative incidence of hip migration increased progressively with age, reaching 60% (CI 55–64) by 16 years of age (Table 2). The rise was most pronounced between 3 and 9 years, where the cumulative incidence increased from 14% at 3 years to 52% at 9 years.

**Table 2:** Cumulative incidence with 95% confidence interval (CI) of MP ≥ 30% by age, accounting for competing risk of death. Age was the underlying time scale. “Hips at risk” denotes hips under observation at the beginning of each age interval. “Events” denotes first MP ≥ 30%, and “Censored hips” denotes hips censored before the next interval

Age	Hips at risk, n	Events, n	Censored hips, n	Cumulative incidence (CI) (%)
1	786	2	0	0.3 (0.0–0.6)
2	784	39	6	5.2 (3.7–6.8)
3	737	62	34	13 (11–15)
4	637	76	30	24 (21–30)
5	531	62	37	33 (30–36)
6	428	39	23	39 (36–43)
7	363	24	29	43 (39–47)
8	310	25	23	48 (44–52)
9	262	19	26	52 (48–56)
10	217	7	23	53 (49–57)
11	186	9	24	56 (52–60)
12	150	4	29	57 (53–61)
13	115	5	24	59 (54–63)
14	84	0	21	59 (54–63)
15	63	0	21	59 (54–63)
16	42	1	24	60 (55–64)

MP = migration percentage.

### Primary analysis: overall hip spasticity

Increasing overall hip spasticity was associated with a higher risk of hip migration (MP ≥ 30%) (Table 3). Compared with MAS 0, hips classified as MAS 1–3, MAS 4–6, and MAS 7–9 had increased subdistribution hazards of hip migration, with sHRs of 1.46 (CI 1.05–2.01), 1.77 (CI 1.22–2.58), and 2.53 (CI 1.59–4.02), respectively.

**Table 3:** Association between hip spasticity and risk of hip MP ≥ 30% accounting for competing risk of death. Subdistribution hazard ratios (sHRs) were estimated using Fine–Gray competing risk regression with death as a competing event, adjusted for GMFCS level (IV vs V) and clustered at child level. Reference category: MAS = 0. Muscle-specific models were fitted separately. Overall hip MAS was defined as the summed MAS score across hip adductors, flexors, and extensors and grouped as 0, 1–3, 4–6, and 7–9

MAS	sHR (CI)	P value
Overall hip MAS		
1–3	1.46 (1.05–2.01)	0.02
4–6	1.77 (1.22–2.58)	0.003
7–9	2.53 (1.59–4.02)	< 0.001
Adductors MAS		
1	1.44 (1.07–1.93)	0.02
2	1.95 (1.33–2.86)	< 0.001
≥ 3	1.97 (1.35–2.87)	< 0.001
Flexors MAS		
1	1.08 (0.84–1.39)	0.6
2	0.88 (0.52–1.49)	0.6
≥ 3	1.86 (1.11–3.14)	0.02
Extensors MAS		
1	1.14 (0.86–1.51)	0.4
2	1.47 (0.95–2.27)	0.09
≥ 3	1.53 (1.01–2.31)	0.05

CI = 95% confidence interval; MAS = Modified Ashworth Scale;

MP = migration percentage (see Figure 2).

### Muscle-specific spasticity

For adductor MAS, all categories above 0 were associated with increased subdistribution hazards, with increased risk already at MAS 1 (sHR 1.44, CI 1.07–1.93) and higher estimates for MAS 2 (sHR 1.95, CI 1.33–2.86) and MAS ≥ 3 (sHR 1.97, CI 1.35–2.87) (see Table 3). In contrast, findings for flexor and extensor MAS were less consistent. For hip flexors, only severe spasticity (MAS ≥ 3) was associated with increased risk of hip migration (sHR 1.86, CI 1.11–3.14), whereas lower MAS categories were not significantly associated. For hip extensors, severe spasticity (MAS ≥ 3) was associated with increased risk (sHR 1.53, CI 1.01–2.31), while MAS 1 and MAS 2 were not statistically significant.

## Discussion

We aimed to investigate, in a register-based cohort study, whether assessed hip spasticity was associated with hip displacement risk in non-ambulant children with CP classified as GMFCS levels IV–V. We found that higher clinically assessed hip spasticity was associated with an increased risk of hip migration, defined as MP ≥ 30%, when accounting for death as a competing event. This association remained after adjustment for GMFCS level (IV vs V), indicating that the observed relationship was not merely driven by differences in gross motor severity within this non-ambulant population. Increasing overall hip spasticity—defined as the summed MAS score across hip muscle groups—was associated with progressively higher subdistribution hazards of hip migration, with a clear dose–response pattern across MAS categories. These results should be interpreted as category-specific comparisons with MAS = 0 rather than as formal evidence of differences between individual MAS levels. Hip adductor spasticity showed the most consistent association with MP ≥ 30% in the mutually adjusted model including adductors, flexors, and extensors, whereas associations observed for hip flexors and extensors in muscle-specific analyses were attenuated or no longer consistent after mutual adjustment. Overall, these findings indicate that the relationship between clinically assessed spasticity and hip migration differs across periarticular hip muscle groups.

Previous studies have suggested that increased muscle overactivity and altered muscle balance around the hip joint may contribute to progressive hip displacement in children with cerebral palsy [6,7]. In line with these biomechanical concepts, the present findings demonstrate an association between higher clinically assessed hip spasticity and an increased risk of hip migration in children with severe functional impairment.

In the mutually adjusted analysis including hip adductor, flexor, and extensor spasticity in the same model, hip adductor spasticity remained independently associated with the risk of hip migration. In contrast, the associations observed for hip flexor and extensor spasticity in the muscle-specific analyses were attenuated and no longer statistically significant after mutual adjustment. The difference between the separate muscle-specific models and the mutually adjusted model suggests that the association with hip migration was most robust for adductor spasticity, whereas associations for flexor and extensor spasticity were less independent after accounting for overlap between muscle groups.

This finding suggests that hip adductor spasticity may be more closely associated with risk of hip migration than spasticity in other periarticular hip muscles. Increased tone in the adductors and flexors is biomechanically more consistent with the classic flexion–adduction “spastic hip position” than increased extensor tone. In contrast, increased extensor tone would not be expected to contribute to lateral displacement through the same mechanism and may in theory be more relevant to anterior displacement, which is not captured by the Reimers migration index. The findings for extensor spasticity should therefore be interpreted more cautiously. This interpretation is broadly consistent with previous biomechanical and imaging-based observations [6,12,18].

The lack of a consistent association for hip flexor spasticity across analyses indicates that the relationship between muscle tone and hip migration is not uniform across muscle groups. In the muscle-specific competing risk analyses, no increased risk of hip migration was observed for mild or moderate flexor spasticity, whereas severe flexor spasticity (MAS ≥ 3) was associated with an increased risk. In the mutually adjusted model, flexor MAS 2 was associated with a lower subdistribution hazard, while severe flexor spasticity was no longer statistically significant.

This pattern suggests heterogeneity in the role of hip flexor spasticity and supports a muscle-specific rather than generalized interpretation of spasticity in relation to hip migration. Given the absence of a consistent dose–response relationship and the counterintuitive finding for moderate flexor spasticity in the mutually adjusted model, these results should be interpreted cautiously and considered hypothesis-generating.

Our findings may have implications for hip surveillance in children with severe cerebral palsy. Current surveillance programs primarily stratify risk based on age and gross motor function, with limited incorporation of muscle-specific clinical findings beyond general tone or contracture assessment [10,15,16]. Within this high-risk population, clinically assessed spasticity—particularly adductor spasticity assessed at the individual hip level—may provide complementary information to existing surveillance approaches. The consistent association between adductor spasticity and MP ≥ 30% is in line with established biomechanical and clinical concepts regarding adductor muscle imbalance in the pathogenesis of hip displacement [6,7,12]. However, spasticity assessment should not be interpreted as a replacement for established surveillance principles, but rather as a potentially useful adjunct in identifying hips that may warrant closer radiographic attention [14,19].

At the same time, the heterogeneous patterns observed across muscle groups underscore that spasticity should not be interpreted as a uniform risk factor. The non-linear associations observed for hip flexor spasticity indicate that muscle-specific functional roles may influence hip migration differently, and that increased tone does not uniformly translate into increased migration risk. This supports a muscle-specific rather than generalized interpretation of spasticity in clinical evaluation

### Limitations

Spasticity was assessed clinically using the MAS, which has demonstrated variable inter- and intra-rater reliability in children with spastic CP [8,20]. Although MAS is widely used in clinical practice and surveillance programs, it represents a subjective assessment and may not fully capture dynamic muscle behavior or functional muscle activity. To address this, spasticity was defined using a landmark approach based on the most recent clinical assessment prior to event or censoring; however, temporal changes in muscle tone during follow-up could not be accounted for. As MAS was frequently assessed close to the time of radiographic evaluation, it may in some cases reflect evolving clinical severity rather than functioning solely as an early independent predictor. In a sensitivity analysis using the first MAS recorded before event or censoring, the associations were weaker and less consistent, supporting that the main findings were most strongly linked to the assessment closest to the radiographic outcome.

Analyses were conducted at the hip level. Although each child could contribute up to 2 hips, within-child correlation was accounted for using cluster-robust standard errors. The results should therefore be interpreted as hip-specific associations rather than child-level effects. Although passive hip ROM data was available descriptively, MAS may not fully distinguish spasticity from fixed contracture, and reduced passive hip abduction and extension may also contribute to altered hip joint forces and hip instability. MAS = 0 may also have included hips without increased tone, including possible hypotonia, as hypotonia could not be identified separately in the available data. Hip displacement may occur in such hips, which should be considered when interpreting spasticity-based risk markers. Additional descriptive analyses did not show a clear monotonic increase in overall summed MAS or adductor MAS with age alone, although higher MAS scores may still in some cases reflect fixed contracture rather than pure spasticity.

Information on surgical or non-surgical interventions targeting the hip or periarticular muscles was not available in the dataset. In clinical practice, reconstructive hip surgery is rarely performed before the chosen threshold MP ≥ 30%, which reduces the likelihood that surgical treatment substantially influenced the primary outcome definition in the present study [15,16]. However, non-surgical tone-modifying interventions, such as botulinum toxin injections or other spasticity treatments, may have been administered prior to MP ≥ 30% and could not be accounted for. Such interventions may influence the progression of hip migration and therefore represent potential unmeasured confounders. Consequently, the observed associations reflect spasticity as recorded in routine clinical practice rather than an untreated natural history.

Finally, the study population was restricted to children classified as GMFCS levels IV–V, which limits generalizability to children with milder functional impairment. This restriction was intentional, as we aimed to evaluate muscle-specific spasticity within a population already at high risk of hip migration.

### Conclusion

In children with CP classified as GMFCS levels IV–V, higher clinically assessed hip spasticity was associated with an increased risk of hip migration. Hip adductor spasticity demonstrated the most consistent association with hip migration, whereas associations for other muscle groups were more heterogeneous. These findings suggest that muscle-specific assessment of hip spasticity may provide complementary information in hip surveillance for children at high risk of migration.

### Supplementary data

Tables 4–8 and Figure 3 are available as Supplementary data on the article homepage, doi: 10.2340/17453674.2026.46169

## Supplementary Material



## Notes

MB, AI, MMP, and ABC designed the study. MB and AI finished data analysis under the direction of ABC. All authors contributed to the data interpretation. MB wrote the first draft, and all authors contributed to revising and acceptance of the final manuscript.

Handling co-editors: Ilkka Helenius and Robin Christensen

*Acta* thanks Freeman Miller, Unni Narayanan, and other anonymous reviewers for help with peer review of this manuscript.
