# Supplementary material for: Association between spasticity of the hip and development of hip displacement in children: a cohort study of 786 hips
Source: Acta Orthop. 2026 Jun 22;97:417–22. doi: 10.2340/17453674.2026.46169 (PMC13285363; doi:10.2340/17453674.2026.46169)
Supplement: Supplementary file 1 [file ActaO-97-46169-s1.pdf]

Supplementary data

Table 4. Mutually adjusted association between muscle-specific hip spasticity and risk of hip migration ≥ 30%, accounting for competing risk of death. Subdistribution hazard ratios (sHRs) were estimated using Fine–Gray competing risk regression with death as a competing event. Adductor, flexor, and extensor MAS were entered simultaneously, adjusted for GMFCS level (IV vs V), and clustered at child level. Reference category: MAS = 0 for each muscle group

MAS	sHR	P value
Adductors MAS		
1	1.52 (1.09–2.11)	0.01
2	2.13 (1.37–3.33)	< 0.001
≥ 3	1.90 (1.21–3.00)	0.006
Flexors MAS		
1	0.81 (0.60–1.10)	0.2
2	0.53 (0.29–0.98)	0.04
≥ 3	1.16 (0.60–2.27)	0.7
Extensors MAS		
1	1.05 (0.76–1.45)	0.8
2	1.30 (0.77–2.20)	0.3
≥ 3	1.21 (0.67–2.19)	0.5

Table 5. Passive hip range of motion (ROM) at the time of MAS assessment. Values are mean (SD). Passive hip ROM measures were recorded during the same standardized clinical assessment as the MAS

Status	Hips, n	Hip abduction	Hip flexion	Hip internal rotation	Hip external rotation	Hip extension
Censored	387	37 (14)	124 (18)	53 (15)	52 (15)	9 (11)
Dead	25	37 (14)	121 (20)	46 (16)	53 (13)	6 (17)
MP ≥ 30%	374	38 (14)	125 (16)	54 (16)	53 (15)	8 (11)

Table 6. Age-specific comparison of spasticity scores in hips with MP < 30% and MP ≥ 30%. Values are mean (SD). Age refers to age in completed years at the relevant radiographic assessment. Overall hip MAS was defined as the sum of MAS scores for hip adductors, flexors, and extensors. Values are count or mean MAS (standard deviation)

Age	MP < 30%, n	Overall hip MAS	Adductors MAS	MP ≥ 30%, n	Overall hip MAS	Adductors MAS
0	2	2.00 (1.41)	0.50 (0.71)	2	4.50 (6.36)	1.50 (2.12)
1	26	2.15 (2.09)	1.00 (1.02)	39	3.70 (2.84)	1.58 (1.20)
2	48	1.50 (1.85)	0.58 (0.77)	62	2.66 (2.05)	1.25 (1.01)
3	40	1.77 (2.38)	0.89 (1.26)	76	2.91 (2.21)	1.35 (0.97)
4	40	2.38 (2.49)	0.95 (1.13)	62	2.67 (2.18)	1.23 (1.15)
5	28	2.68 (2.09)	1.18 (0.94)	39	3.44 (2.31)	1.49 (1.07)
6	32	1.56 (2.02)	0.66 (0.79)	24	2.63 (2.28)	0.83 (0.76)
7	41	1.71 (1.93)	0.63 (0.73)	25	3.71 (3.04)	1.48 (1.19)
8	52	2.22 (1.71)	1.08 (1.01)	19	1.39 (1.42)	0.61 (0.78)
9	26	2.77 (2.73)	1.08 (1.16)	7	2.71 (3.15)	0.86 (1.07)
10	38	1.71 (1.84)	0.63 (0.75)	9	4.44 (3.84)	1.56 (1.24)
11	12	2.00 (1.28)	0.75 (0.97)	4	1.25 (1.26)	1.00 (0.82)
12	14	2.93 (2.16)	1.43 (1.09)	5	1.75 (0.96)	0.80 (0.84)
13	5	4.60 (1.67)	1.80 (0.84)	–	–	–
14	6	1.67 (1.37)	0.50 (0.55)	–	–	–
15	2	2.50 (2.12)	1.50 (2.12)	1	0.00 (NA)	0.00 (NA)

NA = not applicable.

Table 7. Sensitivity analysis using MP ≥ 40% as the outcome. Subdistribution hazard ratios (sHRs) were estimated using Fine–Gray competing risk regression with death as a competing event, adjusted for GMFCS level (IV vs V) and clustered at child level. Overall hip MAS was grouped as 0, 1–3, 4–6, and 7–9

MAS	sHR (CI)	P value
Overall hip MAS		
1–3	1.64 (1.03–2.61)	0.04
4–6	2.07 (1.22–3.49)	0.007
7–9	2.90 (1.53–5.48)	0.001
Adductors MAS		
1	1.67 (1.10–2.53)	0.02
2	2.01 (1.25–3.23)	0.004
≥ 3	2.57 (1.56–4.23)	0.0002
Flexors MAS		
1	1.02 (0.73–1.44)	0.9
2	0.81 (0.43–1.50)	0.5
≥ 3	1.78 (0.90–3.52)	0.1
Extensors MAS		
1	1.09 (0.75–1.57)	0.7
2	1.42 (0.85–2.38)	0.2
≥ 3	1.30 (0.73–2.34)	0.4

Table 8. Sensitivity analysis using first MAS before event or censoring. Subdistribution hazard ratios (sHRs) were estimated using Fine–Gray competing risk regression with death as a competing event. All models were adjusted for GMFCS level (IV vs V) and clustered at child level. Overall hip MAS was defined as the summed MAS score for hip adductors, flexors, and extensors and grouped as 0, 1–3, 4–6, and 7–9

MAS	sHR (CI)	P value
Overall hip MAS		
1–3	0.99 (0.72–1.36)	0.9
4–6	1.08 (0.73–1.59)	0.7
7–9	1.18 (0.76–1.84)	0.5
Adductors MAS		
1	0.98 (0.72–1.35)	0.9
2	1.47 (1.00–2.15)	< 0.05
≥ 3	1.04 (0.71–1.53)	0.8
Flexors MAS		
1	0.92 (0.70–1.22)	0.6
2	1.15 (0.66–1.98)	0.6
≥ 3	1.11 (0.70–1.78)	0.7
Extensors MAS		
1	1.23 (0.90–1.68)	0.2
2	1.24 (0.82–1.88)	0.3
≥ 3	1.44 (0.97–2.15)	0.07

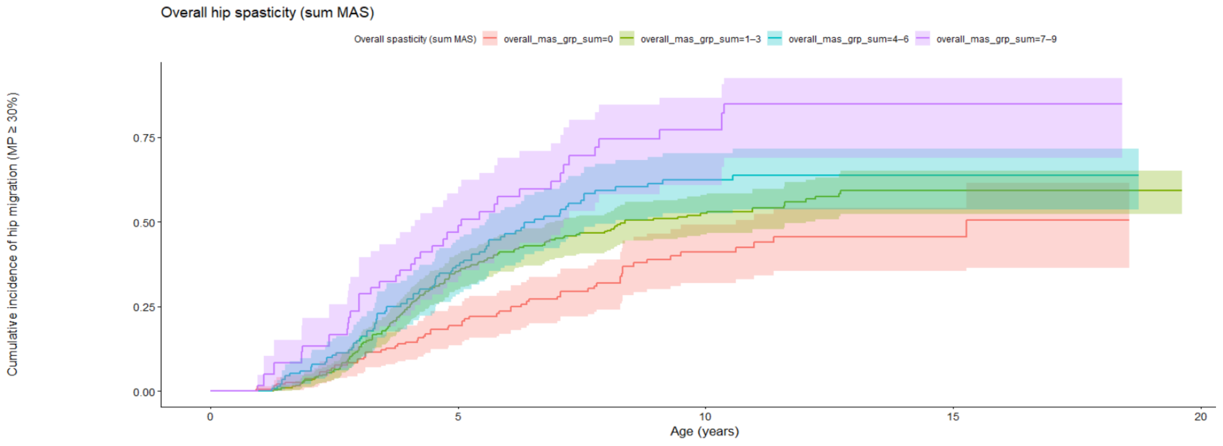
Overall hip spasticity (sum MAS)
Overall spasticity (sum MAS)
overall_mas_grp_sum=0
overall_mas_grp_sum=1–3
overall_mas_grp_sum=4–6
overall_mas_grp_sum=7–9
Cumulative incidence of hip migration (MP ≥ 30%)
0.00
0.25
0.50
0.75
0
5
10
15
20
Age (years)

S2

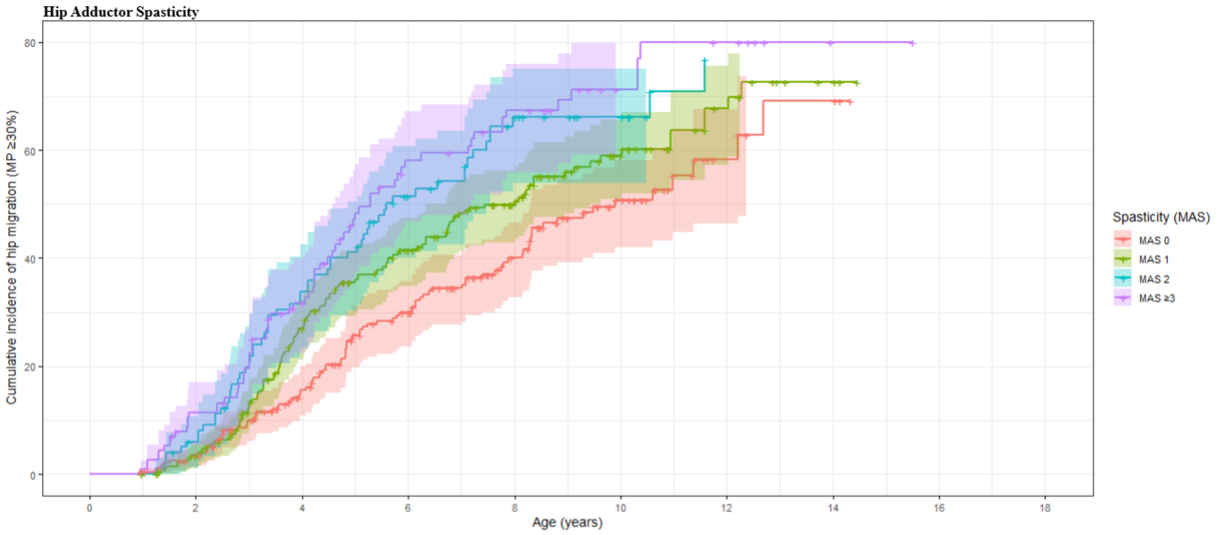
Hip Adductor Spasticity
Cumulative incidence of hip migration (MP ≥30%)
0
20
40
60
80
0
2
4
6
8
10
12
14
16
18
Age (years)
Spasticity (MAS)
MAS 0
MAS 1
MAS 2
MAS ≥3

S3

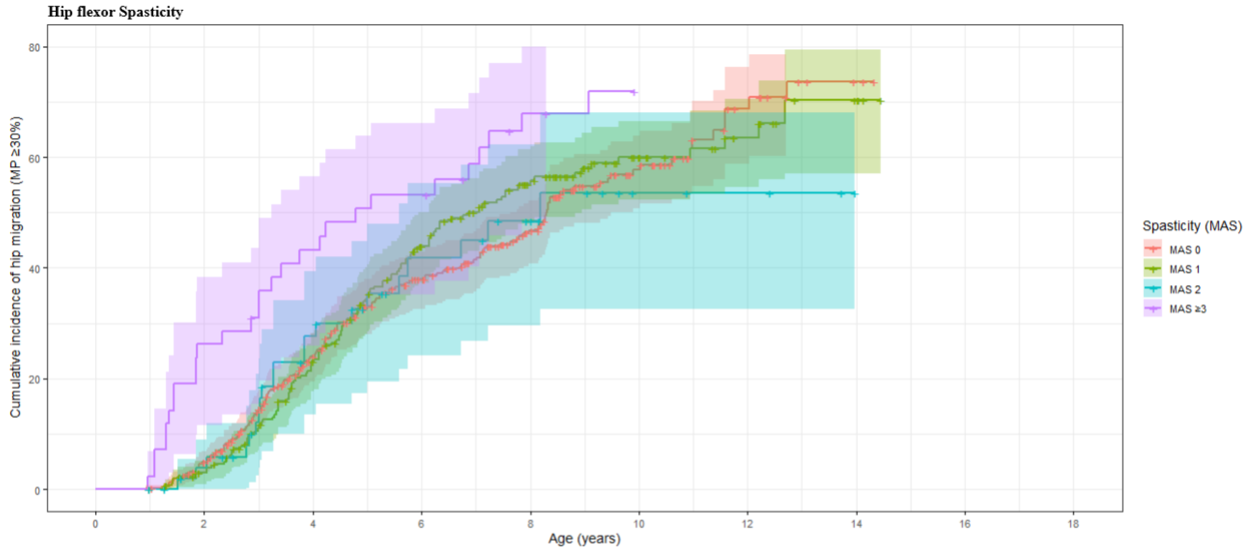

S4

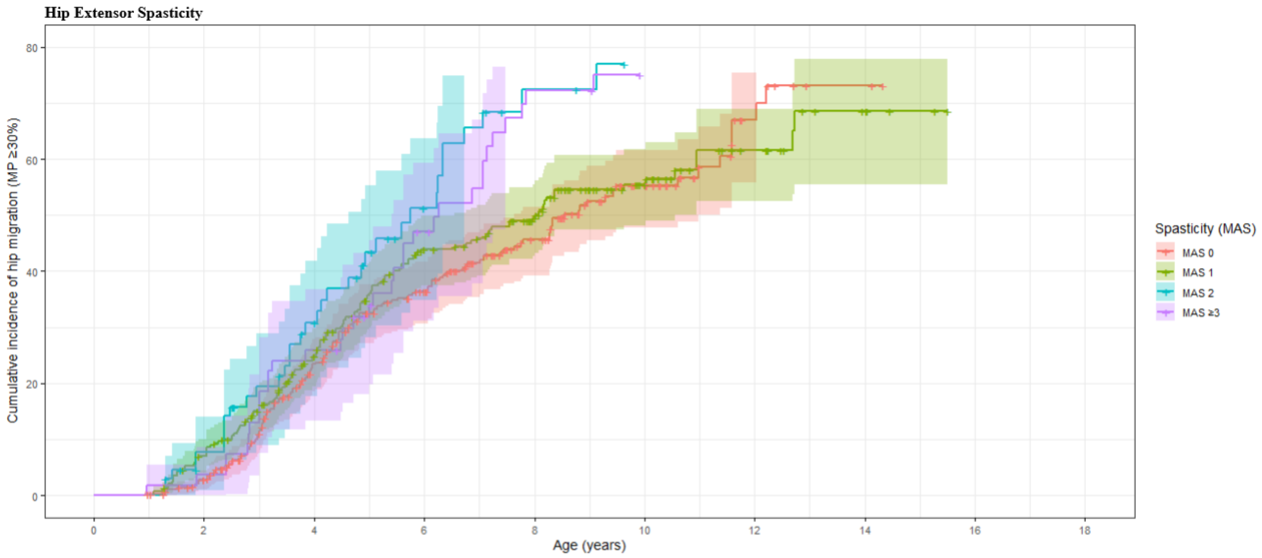

S5

Figure 3. Cumulative incidence curves for MP ≥ 30% by hip spasticity category. Cumulative incidence curves using age as the time scale. Panels show overall hip MAS (S2), adductor MAS (S3), flexor MAS (S4), and extensor MAS (S5). Hips were followed until MP ≥ 30% or censoring at the last radiograph.
